# ECT-induced cognitive side effects are associated with hippocampal enlargement

**DOI:** 10.1038/s41398-021-01641-y

**Published:** 2021-10-08

**Authors:** Miklos Argyelan, Todd Lencz, Simran Kang, Sana Ali, Paul J. Masi, Emily Moyett, Andrea Joanlanne, Philip Watson, Sohag Sanghani, Georgios Petrides, Anil K. Malhotra

**Affiliations:** 1 Psychiatry Research, The Zucker Hillside Hospital, Glen Cove, NY USA; 2 Institute of Behavioral Science, Feinstein Institutes for Medical Research, Manhasset, NY USA; 3 Donald and Barbara Zucker School of Medicine at Hofstra/Northwell, Hempstead, NY USA

**Keywords:** Predictive markers, Hippocampus

## Abstract

Electroconvulsive therapy (ECT) is of the most effective treatments available for treatment-resistant depression, yet it is underutilized in part due to its reputation of causing cognitive side effects in a significant number of patients. Despite intensive neuroimaging research on ECT in the past two decades, the underlying neurobiological correlates of cognitive side effects remain elusive. Because the primary ECT-related cognitive deficit is memory impairment, it has been suggested that the hippocampus may play a crucial role. In the current study, we investigated 29 subjects with longitudinal MRI and detailed neuropsychological testing in two independent cohorts (*N* = 15/14) to test if volume changes were associated with cognitive side effects. The two cohorts underwent somewhat different ECT study protocols reflected in electrode placements and the number of treatments. We used longitudinal freesurfer algorithms (6.0) to obtain a bias-free estimate of volume changes in the hippocampus and tested its relationship with neurocognitive score changes. As an exploratory analysis and to evaluate how specific the effects were to the hippocampus, we also calculated this relationship in 41 other areas. In addition, we also analyzed cognitive data from a group of healthy volunteers (*N* = 29) to assess practice effects. Our results supported the hypothesis that hippocampus enlargement was associated with worse cognitive outcomes, and this result was generalizable across two independent cohorts with different diagnoses, different electrode placements, and a different number of ECT sessions. We found, in both cohorts, that treatment robustly increased the volume size of the hippocampus (Cohort 1: *t* = 5.07, Cohort 2: *t* = 4.82; *p* < 0.001), and the volume increase correlated with the neurocognitive *T*-score change. (Cohort 1: *r* = −0.68, *p* = 0.005; Cohort 2: *r* = −0.58; *p* = 0.04). Overall, our research indicates that novel treatment methods serving to avoid hippocampal volume increase may result in a better side effect profile.

## Introduction

Electroconvulsive therapy (ECT) is a highly effective treatment for multiple psychiatric disorders including treatment-resistant depression [1], bipolar disorder [2], and schizophrenia [3–5]. Nevertheless, it is significantly underutilized [6, 7] perhaps in part due to its association with occasional, but potentially severe, cognitive side effects [8–11]. ECT treatment has been linked to decrements in anterograde and retrograde memory, attention, and executive function, with impairments persisting up to 6 months following completion of a course of ECT [11, 12]. Of note, there is great variability in the occurrence and severity of ECT-induced cognitive side effects. The majority of patients suffer no to minimal effects, whereas a few patients may display marked impairments following a course of treatment. Moreover, the risk of cognitive side effects is independent of the underlying diagnosis of the patient, and there is little data on the mechanism by which ECT induces cognitive side effects.

Because of the potential for ECT to induce marked impairments in memory, the hippocampus has been implicated in ECT treatment effects. While both animal and human neuroimaging studies indicate that ECT (ECS in animals) has a disproportionate cellular and volumetric effect on the hippocampus compared to other brain areas, the clinical relevance of these findings remains controversial. Dukart et al. showed that hippocampal enlargement in unipolar and bipolar depression was associated with clinical improvement, however, the sample size was small (*n* = 5 per group), respectively. Larger studies by the Global ECT-MRI Research Collaboration [13] (GEMRIC) in 282 individuals did not observe an association between hippocampus volume change and clinical response [14], although cognitive side effects were not examined.

Although hippocampal volume change has not consistently been associated with clinical improvement, it may be more plausible that hippocampal volume change is related to ECT-induced cognitive effects. Indirect evidence for this hypothesis is provided by consistent data suggesting that ECT using right unilateral (RUL) electrode placement [15–17], which induces an electrical field that minimizes the involvement of the hippocampus in the dominant hemisphere [18], is associated with decreased cognitive effects as compared to ECT treatment with bilateral electrode placement (BL). Three small neuroimaging studies provide conflicting evidence on the role of hippocampal enlargement in mediating ECT-induced cognitive side effects. The first such study [19] failed to detect a relationship between hippocampal volume change and cognitive decline in 15 depressed patients undergoing RUL ECT. A second study [20] of 12 depressed patients with varying ECT placements also failed to support a relationship between hippocampal change and cognitive deficits. On the other hand, a study of 19 patients with MDD undergoing a lengthy course of ECT with bitemporal electrode placement reported that ECT-induced cognitive side effects were related to the amount of hippocampal enlargement [21]. However, this study did not investigate whether this relationship was hippocampus-specific or was also seen in other areas.

To address this, we have assessed two cohorts of ECT treated patients to understand the relationship between ECT-induced cognitive side effects and hippocampal volume change. First, we examined the relationship between hippocampal volume change and cognition in a cohort of subjects with MDD undergoing ECT with bifrontal electrode placement, by conducting MRI scans at baseline and during treatment. Next, we sought to extend our results by assessing the relationship between ECT-induced cognitive side effects and hippocampal volume change in a cohort of schizophrenia-spectrum disorder patients undergoing ECT with bitemporal placement, again with MRI scans at baseline and at the completion of a course of treatment. We hypothesized that those patients who exhibited the greatest amount of hippocampal enlargement during ECT would demonstrate the greatest degree of cognitive side effects, irrespective of the diagnostic group and electrode placement.

## Patients and methods

The study contains two independent cohorts of patients (*N* = 29) who underwent a longitudinal neuroimaging study during a clinical ECT trial.

**Fig. 1:**
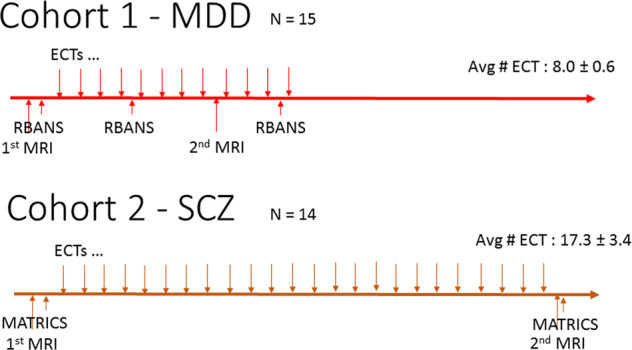
Study design. **a** Cohort 1—MDD (*N* = 15). **b** Cohort 2—SCZ (*N* = 14).

The first cohort consisted of 15 subjects (age: 33.2 ± 11.6 y, 8 F) who received bifrontal only ECT treatment for Major Depressive Episode (baseline HAM-D = 23.1 ± 4.1, Supplementary Table 1). The research team followed the participants for 12 ECT sessions which took place 2–3 times per week initially, and then weekly as the patient improved. The patient’s clinical symptoms were assessed at each ECT with the 24 items Hamilton Depression Rating Scale (HAM-D), and cognitive function was assessed with the Repeatable Battery of the Assessment of Neuropsychological Status (RBANS) at baseline, before the 5th ECT, and before the 12th ECT [22]. In addition, we conducted a brief Mini-Mental Status Exam at each ECT session to rule out critical cognitive decline during the study. Each patient underwent two MRI imaging sessions, at baseline and after the 8th ECT (Fig. 1a).

The second cohort was comprised of 14 subjects (age: 38.8 ± 12.7 y, 4 F) with schizophrenia spectrum disorder (SCZ) (schizophrenia, schizoaffective disorder, schizophreniform disorder) who received bitemporal ECT for medication-resistant psychotic symptoms (baseline BPRS = 42.1 ± 10.0). Patients were treated with ECT 2–3 times/week initially and then 1 time per week as symptoms remitted. Clinical symptoms were assessed with the Brief Psychiatric Rating Scale (BPRS) conducted weekly for the first month of treatment and every 2 weeks thereafter. To assess cognition, the Measurement and Treatment Research to Improve Cognition in Schizophrenia (MATRICS) Consensus Cognitive Battery was conducted at baseline and following 8 weeks of treatment (Fig. 1b) [23].

In parallel with recruiting patients with schizophrenia spectrum disorder, we also recruited age-matched healthy controls to test the practice effect in the MATRICS neurocognitive battery. 29 subjects (age: 31.7 ± 9.1 y, 16 F) were recruited for baseline and 8-week testing.

The RBANS neurocognitive battery measures five cognitive domains: visuospatial, language, attention, immediate, and delayed memory. The MATRICS neurocognitive battery measures seven cognitive domains: speed of processing, attention, working memory, verbal learning, visual learning, reasoning and problem solving, and social cognition. We used the total aggregate scores from both the RBANS (1st cohort) and MATRICS (2nd cohort) batteries to measure the cognitive status of the participants. One individual in the second cohort could not participate in baseline cognitive testing due to severe psychotic symptoms. In addition, we also report the correlation values between volume changes and cognitive scores across all cognitive domains, but our sample is underpowered to conduct any statistical testing in these subdomains (Supplementary Tables 2 and 3). Since the RBANS uses the standard score (mean ± sd: 100 ± 15) and the MATRICS *T*-scores ((mean ± sd: 50 ± 10) to standardize measurements we converted RBANS standard scores to T-scores to be able to compare them across cohorts. All participants provided written informed consent before participation which was approved by the Institutional Review Board of the Feinstein Institutes for Medical Research, Manhasset, NY.

### ECT sessions

All ECT treatments were performed at The Zucker Hillside Hospital ECT unit with similar protocols. All of the 15 patients in the first cohort received ECT with bifrontal electrode placement. Initially, we determined the administered dose based on the half age method. All 14 patients in the second cohort received ECT with bitemporal electrode placement. In this cohort, we determined the seizure threshold on the first session (with 5%, 10%, 20% steps of the maximum) and used 150% of the seizure threshold from the second session on. We increased dosage with 150% increments over the period of ECT treatment if seizure length was reduced under 20 s. None of the patients needed anesthesia agent change during the study due to inefficient seizures. All patients received 1 mg/kg methohexital iv as an anesthesia induction agent and 1 mg/kg succinylcholine for muscle relaxation. Patients who were taking benzodiazepines received 0.2 mg flumazenil shortly before the treatment to counteract the anti-seizure effect of the benzodiazepines. All patients were asked to continue medications as they were taking it before the treatment, except mood stabilizers which we tapered off for the ECT trial. We used a Thymatron device (Somatics, Lake Bluff, IL, USA) to administer ECT. The device delivers a current of 900 mA with a maximum of 504 mC charge. Both bifrontal and bitemporal electrode placements deliver 1 ms wide biphasic square waves with sliding scale frequency between 30 and 70 Hz to keep stimulation duration low (higher doses are delivered with higher frequency).

The length of the ECT course was different across the studies. While the first cohort had on average 8.0 ECT between MRI images, the second cohort had on average 17.3.

### MRI imaging

MR imaging exams were acquired at the North Shore University Medical Center on a Siemens Prisma 3.0T MRI system with standard procedures (MR safety screening, noise reduction, head support, real-time monitoring, 24hr read from neuroradiology). At each imaging session we obtained both T1-weighted images with high resolution (0.7 mm isotropic) MPRAGE sequence (TR = 2400 ms,TE = 2.14 ms, matrix = 320x320, FOV = 224 mm), producing 256 contiguous images (slice thickness = 0.7 mm) through the whole head, and a T2-weighted Fast Spin Echo (SPACE sequence, TR = 3200 ms, TE = 565ms, 320 × 320 matrix, FOV = 224 mm) with 0.7 mm isotropic resolution.

### Image processing

Images were automatically processed with the longitudinal version of Freesurfer 6.0 (Reuter et al., 2012). This provides a robust and reliable estimation of the subcortical volumes and cortical thickness by creating an unbiased within-subject template image (Reuter and Fischl, 2011) using inverse consistent registration (Reuter et al., 2010). In more detail, we cross-sectionally processed both time points separately with the default Freesurfer workflow and created an unbiased template from both time points for each subject. Once this template is created, parcellations and segmentation are carried out at each time point initialized with common information from the within-subject template (Reuter et al., 2012). We identified 42 bilateral regions (9 subcortical volumes and 33 cortical thickness) and calculated the percent changes in these regions by calculating the 100×(region2−region1)/mean(region2 + region1). Due to the use of bilateral symmetrical electrode placements (BF and BT) and limited sample size we averaged corresponding bilateral brain regions to improve the precision of our calculations.

### Statistical analyses

The primary outcome measures were the change in the cognitive function as assessed by the RBANS or MATRICS battery (*T*-Score differences between time points), the hippocampal volume change as assessed by structural MRI. The first cohort had two follow-up time points with neurocognitive assessment at the 5th and 12th ECT, respectively. As the 2nd MRI was conducted between these two-time points, we used the mean RBANS value as the primary cognitive outcome in the first cohort.

We also tested all brain regions for significant volume change. We used the Benjamini and Hochberg FDR correction for multiple comparison corrections.

Other outcome measures that we explored were the clinical responses measured by the HAM-D (1st cohort) and the BPRS (2nd cohort). Instead of absolute change, the individual rate of change was shown to provide more robust results in previous studies [24]. We modeled the change with and without time as an explanatory variable and compared the log-likelihood ratio of the two models with the Chi-square test [24]. Note that a negative value indicated improving clinical status. In contrast, a negative change in the cognitive scores indicated impairment in cognitive functions.

We used a combination of jupyter python notebooks with numpy and pandas packages and R scripts to analyze data and create figures. These scripts and anonymized data tables are available at http://github.com/argy elan/Publications/tree/master/VOLUMEvsCognition.

## Results

### Cohort #1

#### Clinical and cognitive changes

In the cohort of patients with MDE, clinical symptoms improved significantly: HAM-D rating decreased from 23.1 ± 4.1 to 11.4 ± 5.1 (*χ*^2^ = 15.4, df = 1, *p* = 0.00009) (Supplementary Fig. 1A). RBANS total score also decreased by 2.4 points (range: −10 to 7, *t* = 1.58, df = 14, *p* = 0.14) after 5 ECT treatments and by 2.0 points (range: −15 to 18, *t* = 0.80, df = 11, *p* = 0.44) after 12 ECT treatments (Supplementary Fig. 2A). These changes were not significant, however 6 out of the 15 patients, despite clinical improvement, experienced more than 7 points (1 quartile) of a decrease in their overall standard cognitive scores after ECT. The cognitive change and the clinical change did not correlate at any time points (cohort 1, 5th ECT: *r* = −0.12, df = 13, *p* = 0.67; 12th ECT: *r* = 0.05, df = 10, *p* = 0.88).

#### Volumetric changes and its relationship with cognitive changes

**Fig. 2:**
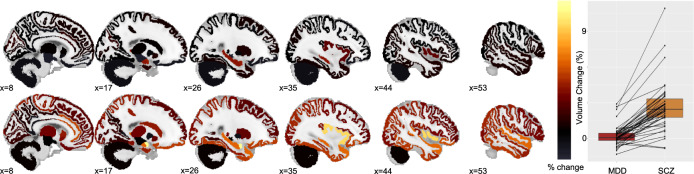
Volume changes across the cortical and subcortical areas. Lower panel: each dot corresponds to one of the 42 regions (for a comprehensive list see Supplementary Tables 2 and 3), similar regions are connected. Color bar represents the average percentage change.

The first cohort showed significant (FDR corrected *p* < 0.05) increases in the hippocampus and the amygdala (Supplementary Table 2, Fig. 2. Upper panel).

##### Hypothesis testing

**Fig. 3:**
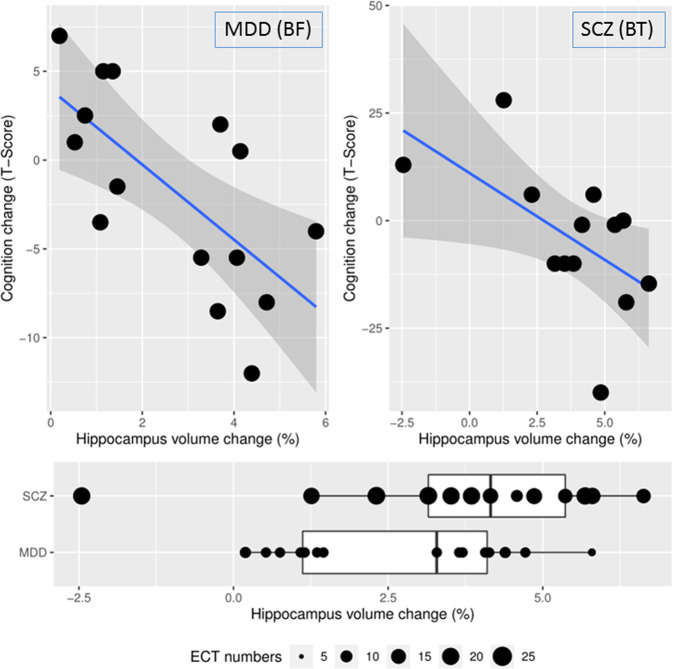
The relationship between hippocampus volume change and cognitive performance change. Left upper panel: Cohort with MDD patients and BF electrode placement, *r* = −0.68, df = 13, *p* = 0.005. Right upper panel: Cohort with schizophrenia patients and BT electrode placements, *r* = −0.58, df = 11, *p* = 0.04, (one individual could not participate in baseline cognitive testing). Lower panel: Patients with depression had on average 8.0 ± 0.6 ECTs between image acquisitions showed an average of 2.68% increase in hippocampal volume, patients with schizophrenia, who had on average 17.3 ± 3.4 ECTs between image acquisitions showed an average of 4.43% increase in hippocampus volume.

There was a significant negative correlation between hippocampal volume change and neurocognitive changes (*r* = −0.68, df = 13, *p* = 0.005) as assessed by the difference between baseline and the mean RBANS score of the two follow-up time points, indicating that greater hippocampal volume increase was associated with lower cognitive performance (Fig. 3 left upper panel). Similar results were obtained using the individual RBANS assessment following the 5th and the 12th ECT treatments (*r* = −0.54, df = 13, *p* = 0.04; *r* = −0.51, df = 10, *p* = 0.09), see Supplementary Fig. 3).

##### Exploratory analysis

We also evaluated the clinical and cognitive correlations in all the other brain regions that survived FDR correction (Supplementary Table 2). In the first cohort, no other areas showed any correlation with clinical or cognitive changes.

### Cohort #2

#### Clinical and cognitive changes

In the second cohort, BPRS ratings indicated significant clinical improvement; the BPRS total score decreased from 42.1 ± 10.0 to 34.2 ± 9.6 (*χ*^2^ = 3.8, df = 1, 0.05) (Supplementary Fig. 1B). MATRICS total *T*-score decreased by 4.35 points (range: −40 to 28, *t* = 0.65, df = 11, *p* = 0.53) after 8 weeks of treatment (Supplementary Fig. 2B). While the cognitive changes were not significant, the results indicated larger individual differences than expected based on neurocognitive data collected in healthy controls. Specifically, healthy controls had an intraclass correlation coefficient (ICC) of 0.79 (95% CI: 0.59–0.90) between baseline and 8th-week measures, demonstrate a consistency of performance across time. By contrast, the ICC observed in the patients across timepoints was 0.28 (95% CI: −0.30 to 0.72). T In addition, healthy controls showed a moderate practice effect (*t* = 2.1, df = 26, *p* = 0.04, Supplementary Fig. 4) that was not observed in the patients.

Similar to the first cohort, the cognitive and clinical effects were not correlated with each other (*r* = −0.05, df = 11, *p* = 0.86).

#### Volumetric changes and its relationship to cognitive changes

In the second cohort, in addition to the hippocampus and the amygdala, there were widespread increases in the volume of temporal lobe structures, such as the superior, middle and inferior temporal gyrus, temporal pole, as well as in the parietal lobe, and the insula, and cortical structures around the insula. (Supplementary Table 3, Fig. 2. Lower panel)

The mean volume change was significantly higher in the second cohort than in the first cohort measured across 42 regions (Fig. 2, right panel, paired *T*-test, *t* = 10.1, df = 41, *p* < 10^−12^). The main difference between these two cohorts was the number of ECTs (the second cohort had a significantly higher number of ECTs, more than double —see the section “Patients and methods”).

##### Hypothesis testing

There was a significant negative correlation between hippocampal volume change and neurocognitive changes (*r* = −0.58, df = 11, *p* = 0.04) indicating that hippocampal volume increase was associated with lower cognitive performance in both cohorts (Fig. 3).

##### Exploratory analysis

We also evaluated the respective clinical and cognitive correlations in all the other areas that survived FDR correction (Supplementary Table 2) in the second cohort. We made two observations. First, hippocampus volume increase correlated with symptomatic improvement (*r* = −0.61, df = 12, *p* = 0.02). Second, other brain region volume increases were positively correlated with cognitive changes, as reflected by the correlation between total gray matter volume increase and cognitive improvement (*r* = 0.53, df = 11, *p* = 0.06). These were exploratory observations and none of these changes survived correction for multiple comparisons.

## Discussion

ECT-induced hippocampal volume increases were significantly correlated with the degree of cognitive impairment induced by ECT. We first observed this relationship in a cohort of patients with depression and then replicated this finding in a second cohort with patients with schizophrenia. These results were consistent despite differences in the patient population and several parameters of the ECT treatment, such as electrode placement and the number of treatments. Most importantly, this is the first demonstration that hippocampus volume change is specifically related to cognitive change, and volume changes in other regions have no or significantly lower association with cognitive side effects. These data strongly support the hypothesis that ECT induces cognitive side-effects via or in conjunction with the effects on the hippocampus.

Many previous studies found micro and macroscopic changes in the hippocampus during electrical stimulation of the brain [14, 25–30], but the specificity and functional relevance of these findings has remained controversial [14, 20, 27–30]. Our results validate the central role of the hippocampus in the cognitive side effects of ECT. The study by Oostrom et al. (2018) has shown similar correlations already but has not investigated other brain regions. Our previous studies indicated that volume enlargement during ECT is widespread across the entire brain [27, 30], therefore it was unclear if the relationship in the Oostrom study only reflected generalized “ECT burden” or region-specific impact. Our study investigated 42 cortical and subcortical regions and our results were specific to the hippocampus. Both the volume increase and the correlation with the cognitive deficits showed selectivity specific to the hippocampus. Hippocampal volume change was 3rd and 5th largest in the two cohorts respectively, and their negative correlations with cognition were the strongest among the regions. These results are especially important in light of growing longitudinal neuroimaging evidence that ECT causes both cortical and subcortical volume changes in a dose-dependent way [30] in close correlation with the magnitude of the applied electric field [27]. Therefore novel methods such as magnetic seizure therapy, which can induce seizures with a less direct effect on the hippocampus, may present a promising new treatment modality to decrease cognitive side effects [31].

We acknowledge that, despite its specificity, this relationship does not necessarily indicate causality. It is well known that depression is associated with a smaller hippocampus [32–35] as well as with impaired cognitive functions [36]. It has also been shown that clinical response is associated with improved cognition [36]. However, both hippocampus enlargement and clinical response during ECT would then implicate better cognitive performance while we have measured the opposite effect. In the same way, schizophrenia patients also show decreased hippocampal volume [37–43] and impaired cognitive functions at baseline [44–47]. However, cognitive functions do not improve with clinical response beyond practice effect [48, 49]. Therefore, the inverse relationship between hippocampus volume change and cognitive function is intriguing and implies a hippocampus-specific biological mechanism behind these cognitive changes.

Based on these results, we can only speculate about the biological underpinnings responsible for the hippocampal volume change. As discussed in detail in our previous report [27], ECT-induced volume changes can be due to fluid shifts due to vascularization [50], blood flow change [51, 52], inflammation [53–56] or vasogenic oedema [57–59] and/or neuroplastic mechanisms including neurogenesis [26, 60–62], synaptogenesis [63, 64] and gliogenesis [65]. Some of these mechanisms are more likely than others given the widespread changes related to and the timescale of the intervention, but more preclinical studies will be required to shed light on the exact nature of these volume changes. This study did not use a healthy control group in the neuroimaging analyses, but the measured volume changes are unlikely to be an artifact or epiphenomena as our previous studies [14, 30] with a similar pipeline indicated very robust structural measures in healthy controls whereas no change was found in the hippocampus between treatments (0.05% ± 0.08%, *N* = 95 [14]) during a similar time interval.

The two independent cohorts of patients were collected in two separate ECT-related studies, therefore there are limitations stemming from the incongruencies between the study designs. One limitation was that the depressed group received significantly fewer treatments than the schizophrenia group given the shorter trial length. Previous studies showed positive correlations between the treatment numbers and the volume changes [14, 27, 30], and we note also that bitemporal electrode placement might cause a more significant impact on the hippocampus by its physical proximity to the anatomical structure. Therefore these group differences were an important confound which lead to measurable differences in the volume changes between the two groups (Figs. 2 and 3). Therefore, we believe it was critical to demonstrate that the mediator effect of the volume change was present independently in both cohorts indicating that this relationship was not driven by group effects or potential confounds. There was also some incongruence between the time of the cognitive batteries and the MRI scan in the depression group. The first group had cognitive assessments three times with approximately 2 weeks time difference, while the latter had assessments only two times with 8 weeks difference. Finally, we used different cognitive batteries in the two cohorts due to time limitations in the first cohort. While the MATRICS battery is somewhat more comprehensive than the RBANS, they both measure overall cognitive abilities across similar cognitive domains and we used this overall total score in both studies as our outcome measure. Despite these limitations and differences between these groups, the replication of results in two independent cohorts with different diagnoses, different electrode placements, and different timelines can be also considered as a strength of the results indicating robust relationships.

In summary, our a priori hypothesis that ECT-induced hippocampal volume increases are associated with cognitive side effects was demonstrated in two independent cohorts. These data suggest that strategies to reduce ECT effects on the hippocampus may be clinically useful and, perhaps, may suggest new strategies to optimize ECT or other forms of convulsive therapies across patient groups.

## Supplementary information

Supplementary Material
