# Supplementary material for: ECT-induced cognitive side effects are associated with hippocampal enlargement
Source: Transl Psychiatry. 2021 Oct 8;11:516. doi: 10.1038/s41398-021-01641-y (PMC8501017; doi:10.1038/s41398-021-01641-y)
Supplement: Supplementary file 1 — Supplementary Material [file 41398_2021_1641_MOESM1_ESM.docx]

# Supplementary Material

## Supplementary Tables

Supplementary Table 1

|  | Cohort 1 | Cohort 2 |
| --- | --- | --- |
| Age | 33.2 ± 11.6 | 38.8 ± 12.7 |
| Sex | 8 F 7 M | 4 F 10 M |
| Diagnosis | 11 MDD and 4 BP (based on clinical history) | SCZ (based on SCID DSM-IV) |
| History of Psychosis | 0/15 | 14/14 |
| Medications (baseline) | 6/15 SSRI; 4/15 SNRI, 4/15 TCA, 8/15 SGAP, 10/15 BDZ | Chlorpromazine equivalent: 593.2 ± 266.2 mg/day  BDZ: 4/14  SSRI:2/14 |
| Medications  (exit) | 6/15 SSRI; 4/15 SNRI, 4/15 TCA, 8/15 SGAP, 10/15 BDZ | Chlorpromazine equivalent: 559.1 ± 247.0 mg/day  BDZ: 3/14  SSRI: 2/14 |
| Clinical Baseline | HAMD: 23.1 ± 4.1 | BPRS: 42.1  ± 10.0 |
| Clinical Exit | HAMD: 11.4 ± 5.1 | BPRS: 34.2 ± 9.6 |
| Number of ECTs | 8.0 ± 0.6 | 17.3 ± 3.4 |
| Days between MRIs | 29.8 ± 6.3 days | 67.5 ± 10.3 days |

*Abbreviations*: MDD: Major Depressive Disorder, BP: Bipolar disorder, SCZ: schizophrenia spectrum disorder, SSRI: selective serotonin reuptake inhibitor, SNRI: serotonin-norepinephrine reuptake inhibitor, TCA: tricyclic antidepressant, SGAP: second generation antipsychotic, BDZ: Benzodiazepines, HAMD: Hamilton Depression Rating Scale, BPRS: Brief Psychiatric Rating Scale

Supplementary Table 2


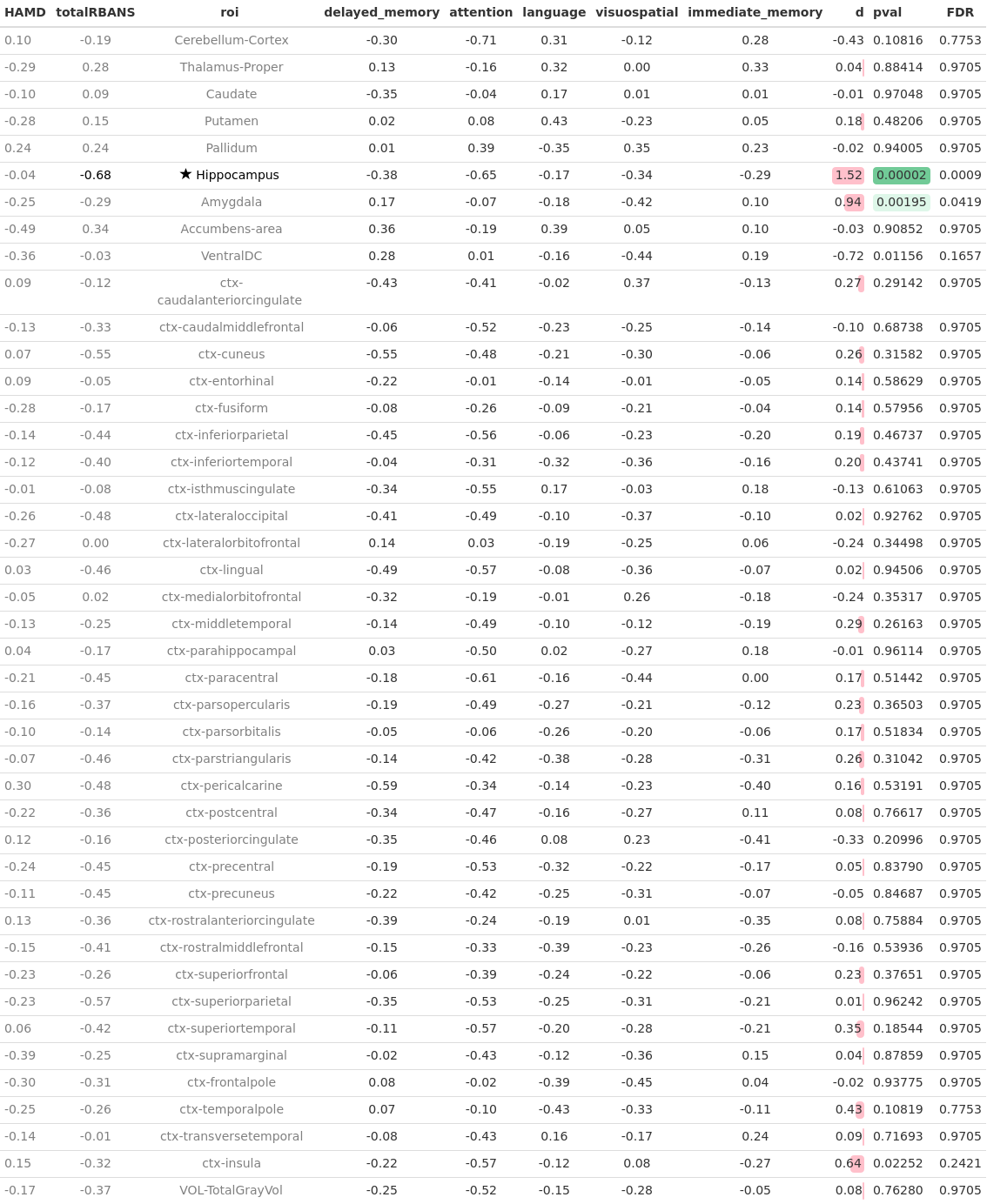


Supplementary Table 3.


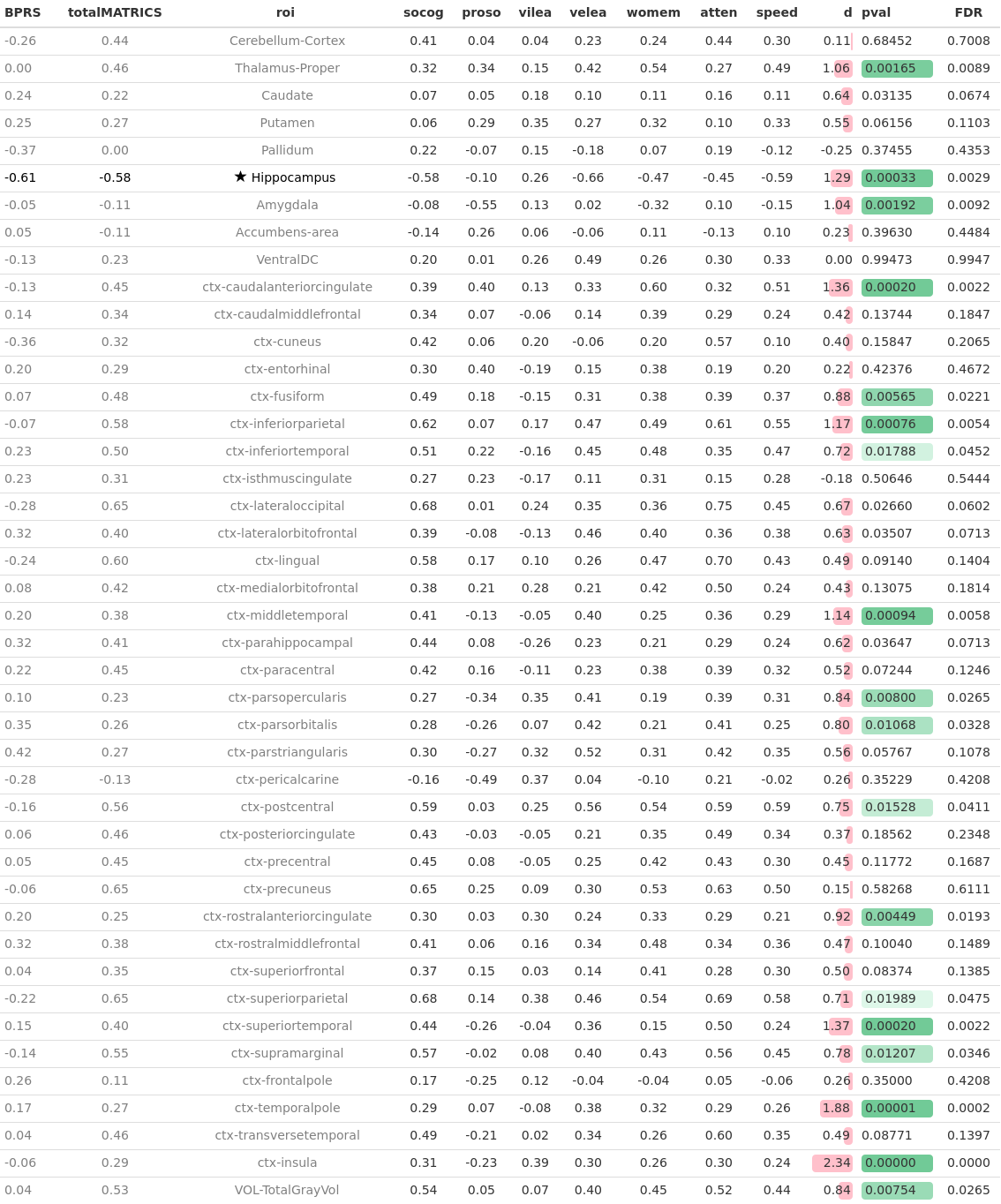


## Supplementary Figures


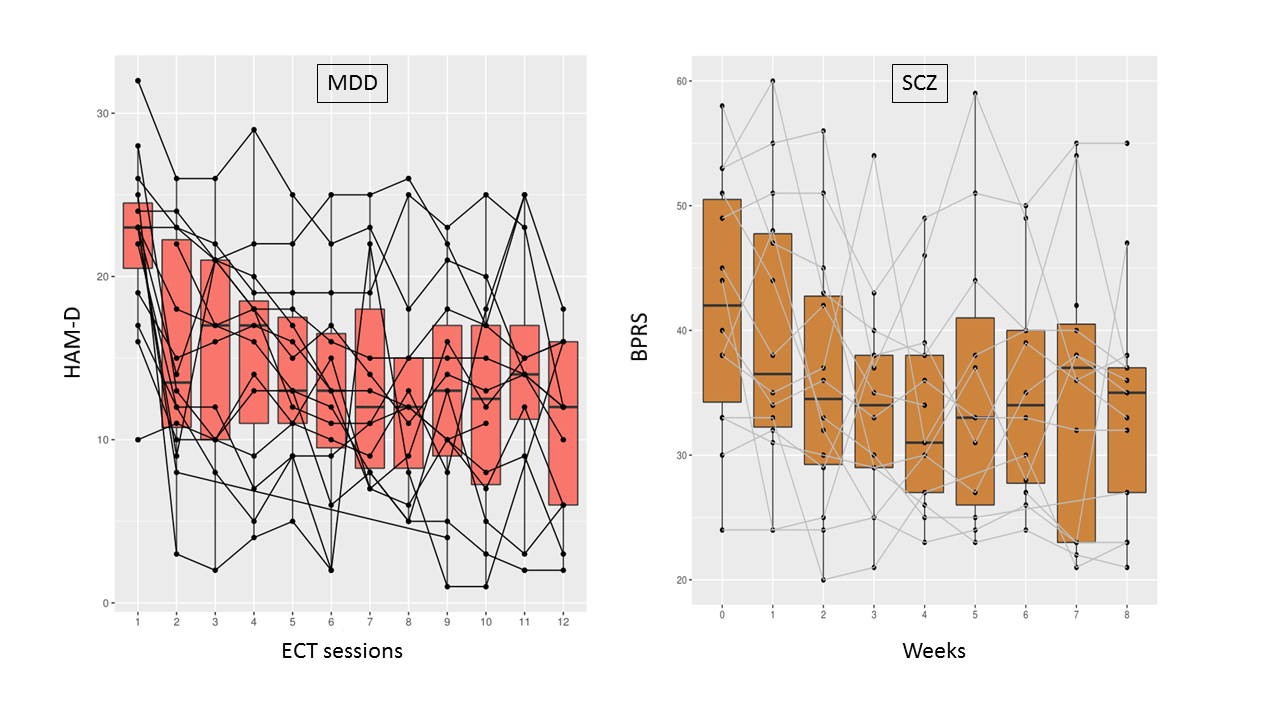


Supplementary Figure 1. Clinical symptoms change during ECT


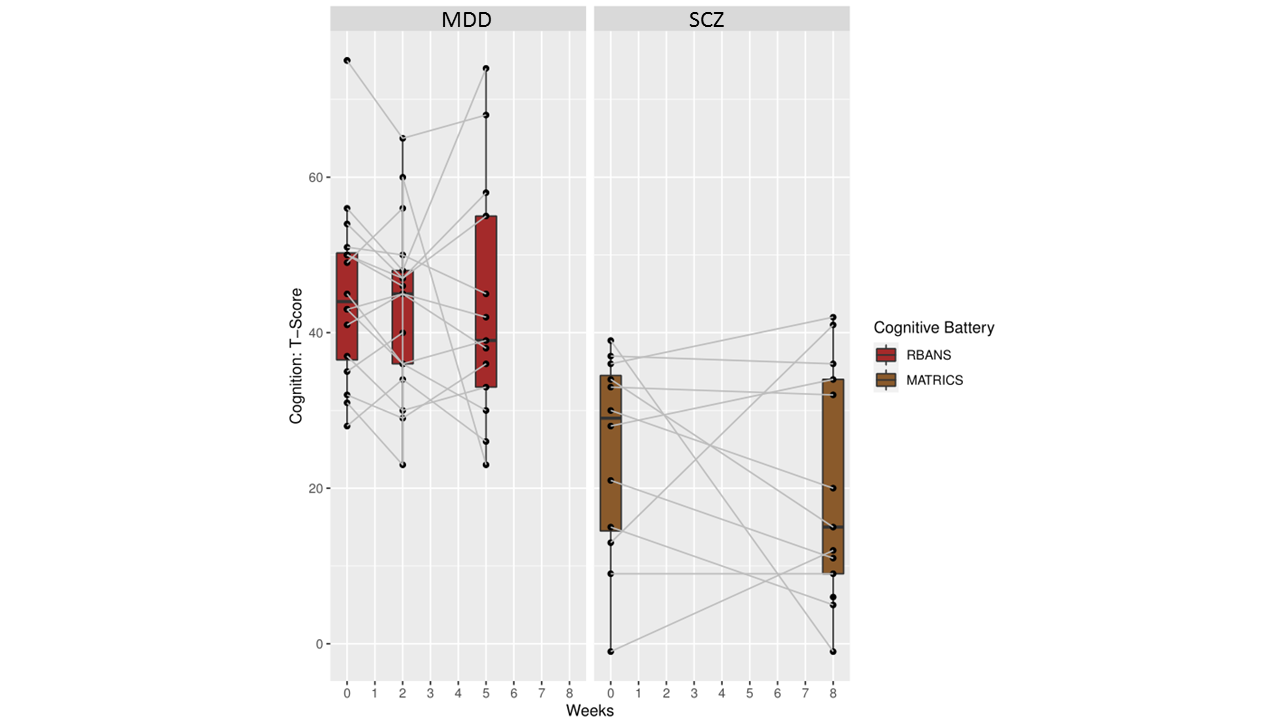

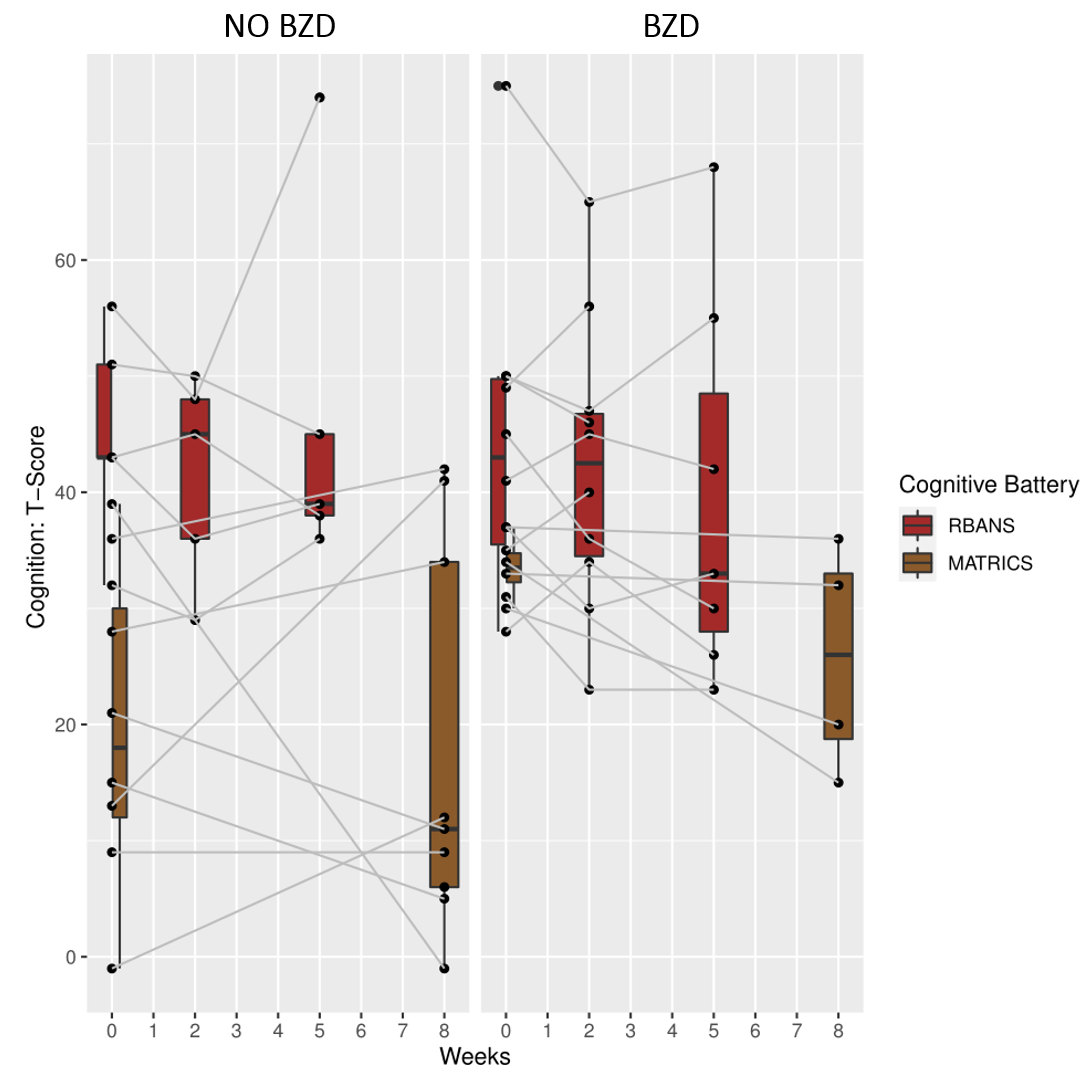


Supplementary Figure 2 A) Cognitive status during ECT. B) Dividing groups based on their benzodiazepine status did not show any medication status group effect (t=0.76, p=0.45) or interaction effect (t=1.6, p=0.11 in the MDD group and t=1.6, p=0.11 in the SCZ group respectively).


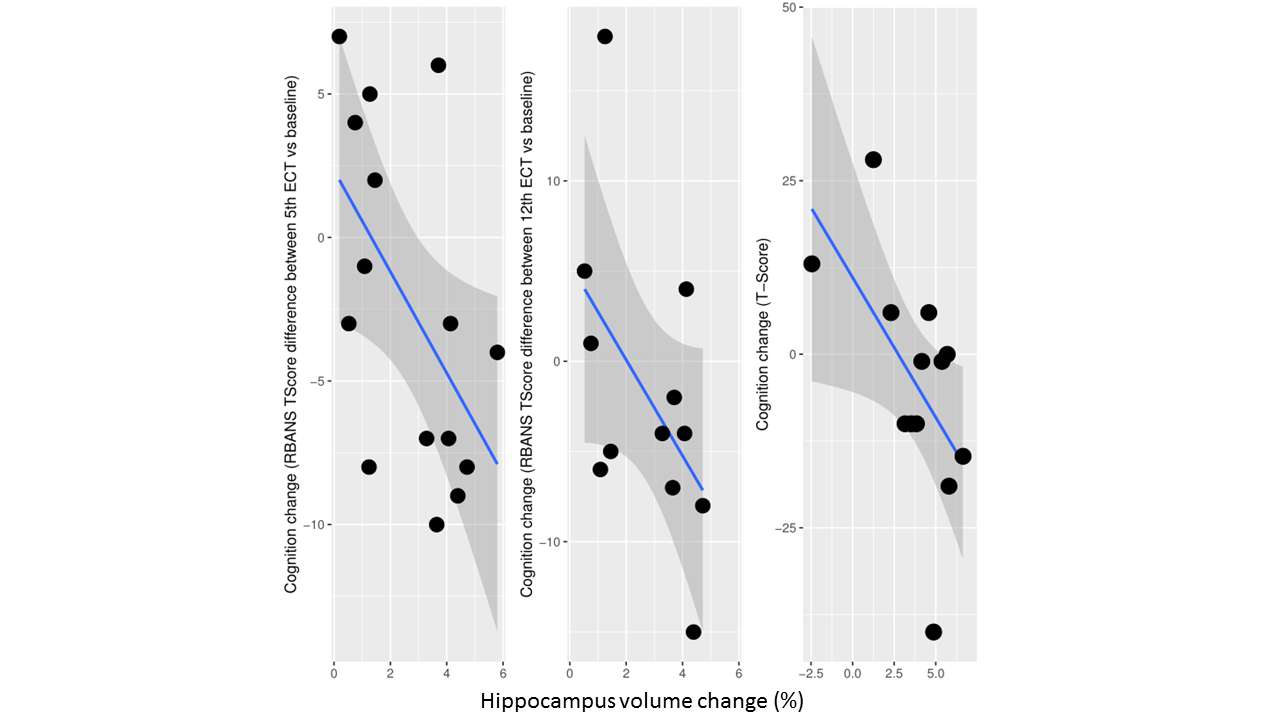


Supplementary Figure 3. The relationship between volume change in the hippocampus and the cognitive changes (showing results at all time points in the first cohort). The left two are from the first cohort (RBANS changes between the 5th ECT and baseline and the 12th ECT and baseline, respectively), the right-sided one is from the second cohort (MATRICS changes between the 8th-week visit and the baseline visit). (LEFT: r=-0.54, df=13, p=0.04, MIDDLE: r=-0.51, df=10, p=0.09, RIGHT: r=-0.58, df=11, p=0.04).


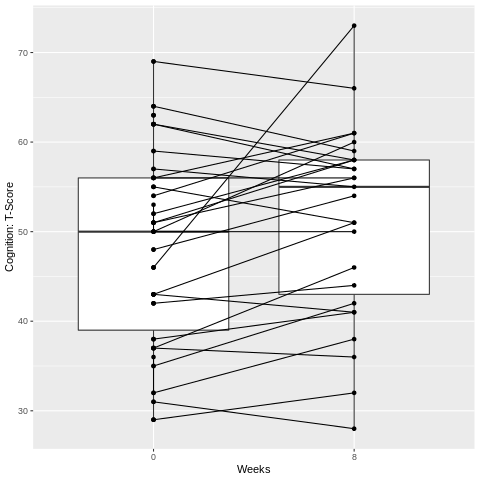


Supplementary Figure 4. Cognitive status in healthy controls

MATRICS battery collected in 29 healthy controls (2 individuals did not have follow up). Paired t-test indicated increased cognitive scores at the second 8-week time point (t=2.1, df=26, p=0.04). Range of change was -5 to 27 score points. The intraclass correlation coefficient (ICC=0.79 with 95% CI: 0.59-0.90) was indicating that measures had good consistency across time points, and the differences were likely due to moderate practice effects.
